# Theabrownin and *Poria cocos* Polysaccharide Improve Lipid Metabolism *via* Modulation of Bile Acid and Fatty Acid Metabolism

**DOI:** 10.3389/fphar.2022.875549

**Published:** 2022-06-27

**Authors:** Jieyi Wang, Dan Zheng, Fengjie Huang, Aihua Zhao, Junliang Kuang, Zhenxing Ren, Tianlu Chen, Jing Lei, Jingchao Lin, Xiaoning Wang, Wei Jia, Guoxiang Xie, Xiaojiao Zheng

**Affiliations:** ^1^ Center for Translational Medicine and Shanghai Key Laboratory of Diabetes Mellitus, Shanghai Jiao Tong University Affiliated Sixth People’s Hospital, Shanghai, China; ^2^ Shenzhen Huiyun Pharmaceutical Technology Co. Ltd., Shenzhen, China; ^3^ Human Metabolomics Institute, Inc, Shenzhen, China; ^4^ Key Laboratory of Liver and Kidney Diseases (Ministry of Education), Institute of Liver Diseases, Shuguang Hospital, Department of Pharmacology, Shanghai University of Traditional Chinese Medicine, Shangha, China; ^5^ Hong Kong Traditional Chinese Medicine Phenome Research Centre, School of Chinese Medicine, Hong Kong Baptist University, Hong Kong, China

**Keywords:** theabrownin, Poria cocos polysaccharide, nonalcoholic fatty liver disease, metabolomics, lipid metabolism

## Abstract

Nonalcoholic fatty liver disease (NAFLD) is prevalent worldwide, while no pharmaceutical treatment has been approved. Natural herbs are promising for their amelioration effect on lipid metabolism. Theabrownin (TB) and *Poria cocos* polysaccharide (PCP) have been reported to have effect on hyperlipidemia and diabetes. Here, we compared the effect of individual TB or PCP and the combination of TB and PCP (TB + PCP) on NAFLD phenotypes and the alteration of metabolism in the mice with high-fat diet. The results showed that TB, PCP, and TB + PCP reduced serum and hepatic lipid levels, among which TB + PCP was the most effective. Serum metabolomic profile and liver mRNA analyses revealed that the treatments altered metabolic pathways involved in fatty acid metabolism, bile acid metabolism, and tricarboxylic acid cycle, which was also most significant in the TB + PCP group. This study demonstrated that TB, PCP, especially the combination of TB and PCP could be potential therapeutic formula for NAFLD that promoted lipid utilization and inhibited lipid synthesis and absorption.

## Introduction

Natural products have been shown to alleviate metabolic diseases such as obesity, diabetes, and nonalcoholic fatty liver disease (NAFLD) by promoting lipolysis (Chen et al., 2021a), lipid transport (Li et al., 2020) and β-oxidation (Chang et al., 2015) in adipose and hepatic tissues (Wu et al., 2019a). At present, many therapeutic candidates are on the clinical trials with good prospectives, such as the agonist of glucagon-like peptide-1, Semaglutide (Negi et al., 2022). However, no pharmaceutical treatment for NAFLD has been approved by international authorities, and natural herbs could be promising alternative remedies (Chen et al., 2017; Yan et al., 2020). The traditional Chinese medicine (TCM) compatibility rationale is regarded as an important theoretical basis on TCM and refers to the inclusion of two or more herbs to form a formula that can obtain more efficient treatment (Zhou et al., 2017). The combination of natural herbs has been explored to protect the liver from NAFLD, cholestasis and hepatocellular carcinoma (Chen et al., 2021b; Zhang, 2015).

Theabrownin (TB) is a kind of water-soluble polyphenols produced through pu-erh tea fermentation (Huang et al., 2019a; Kuang et al., 2020). Our previous study has proved that after TB treatment, the body weight, blood and liver lipid levels are reduced in humans and mice (Huang et al., 2019b). TB could promote cholesterol degradation, insulin sensitivity, energy consumption, and white adipose tissue browning by altering the composition of gut microbiome and bile acid metabolism (Kuang et al., 2020). *Poria cocos*, is a dried sclerotia of *P. cocos* wolf, which is often parasitic on pine roots. *P. cocos* has been reported to be beneficial in the treatment of chronic gastritis, edema, and nephrosis (Sun, 2014). The constituents of *P. cocos* include polysaccharide (PCP), triterpenoid, and steroid, in which PCP is one of the most effective components in anti-inflammatory (Liu et al., 2018; Liu et al., 2021), antioxidant (Wang et al., 2016), hepatoprotection (Wu et al., 2019b), and blood glucose reduction (Wang et al., 2018).

Based on the lipid-lowering effect of TB and PCP, we hypothesized that TB, PCP, or the combination of TB and PCP could alleviate hyperlipidemia. In this study, TB, PCP, and TB + PCP were used to treat NAFLD mice induced by the high-fat diet. The phenotypes including body weight, serum and hepatic lipid levels, glucose tolerance have been tested. We also applied the metabolomic approach on serum samples to evaluate the effect of TB and PCP on lipid and glucose metabolism. The alterations of metabolic pathways were further validated by the mRNA and protein analyses.

## Materials and Methods

### Phytochemical Analysis of TB and PCP

TB was provided by Yunnan Tangren Biological Technology Co., Ltd. (batch number: TB-20130913). The analysis of TB was performed with a system approach (Chen, 1981) by using ultraviolet-visible spectrophotometer (UNIC UV-2102 PCS, UNIC, United States) and completed by China Tea Quality Inspection and Supervision Center.

PCP was provided by Nanjing Zhenweikang Biological Technology Co., Ltd. (batch number: Zwk210317464). The identification of PCP was completed by ShenZhen Kivita Innovative Drug Discovery Institute. Crude polysaccharide was accurately weighed and dissolved in ethanol aqueous solution. After ultrasonic extraction, centrifugation, and precipitation, the content of the crude polysaccharide was determined by phenol-sulphuric acid method using UV-spectrophotometry (Masuko et al., 2005).

### Animal Study

All animal studies were performed following the rules dictated by the national legislation and were approved by the Institutional Animal Care and Use Committee at the Center for Laboratory Animals, Shanghai Jiao Tong University Affiliated Sixth People’s Hospital (DWSY2020-0159). All the mice were maintained in a specific-pathogen-free (SPF) environment with controlled conditions, a 12 h light/dark cycle at 20–22°C and 45 ± 5% humidity.

A total of forty 8-week-old mice were acclimated for 1 week and then were randomly divided into five groups (n = 8 mice per group) and treated with the following diets for 12 weeks: control chow diet (CD), high fat diet (HFD) (the HFD contained 60.9% lipids, 18.3% proteins, and 20.8% carbohydrates), HFD supplemented with 0.3% TB (w/w) (HFD + TB), HFD supplemented with 0.15% PCP (w/w) (HFD + PCP), and HFD with 0.3% TB and 0.15% PCP (w/w) (HFD + TB + PCP). The dose selection of TB and PCP is based on the clinical doses for human, 3 g/day of TB (Huang et al., 2019b) and 1.5 g/day of PCP. All mice were fasted overnight before being euthanized. Samples of intestinal tissue, adipose tissue and liver were carefully collected and kept in liquid nitrogen and then stored at −80°C until analysis.

### Biochemical Analysis

For animal experiments, serum glucose, total cholesterol (TC) and triglycerides (TG) were measured using an automatic biochemical analyzer (Hitachi 7600, Tokyo, Japan). Serum TC, TG (ab285242 and ab65390, respectively, Abcam, Cambridge, United Kingdom), high-density lipoprotein cholesterol (HDL-C), and low-density lipoprotein cholesterol (LDL-C) were measured using a commercial biochemical kit (ab65390, Abcam, Cambridge, United Kingdom). Moreover, the liver TG and TC content were detected using a triglyceride assay kit and total cholesterol assay kit (A110-1-1 and A111-1-1, respectively, Jiancheng Bioengineering Institute, Nanjing, China).

### Insulin Tolerance Test (ITT) and Oral Glucose Tolerance Test (OGTT)

OGTT and ITT were performed after 12 and 4 h of fasting, respectively. About 1 g/kg of glucose and 0.75 units/kg of insulin were given by oral gavage for OGTT test and intraperitoneal injection for ITT, respectively. Blood samples at 0, 15, 30, 60, and 120 min were collected from the caudal vein. The blood glucose levels were measured using a glucose analyzer (OneTouch Ultra, Lifescan, Johnson&Johnson, Milpitas, CA).

### Measurement of Metabolites in Serum

Metabolomics analysis on serum samples (25 μL) was conducted using the Q300 Metabolite Assay Kit (Human Metabolomics Institute, Inc., Shenzhen, Guangdong, China) (Xie et al., 2021a). After sample preparation according to the manufacture’s instruction, the Q300 plate was sealed for ultraperformance liquid chromatography coupled with tandem mass spectrometry (UPLC-MS, ACQUITY UPLC-Xevo TQ-S, Waters Corp., Milford, MA, United States) analysis according to a protocol we previously established. All chromatographic separations were performed with an ACQUITY BEH C18 column (1.7 μm, 100 mm × 2.1 mm internal dimensions; Waters, Milford, MA, United States). The mobile phase consisted of 0.1% formic acid in LC–MS grade water (mobile phase A) and 0.1% formic acid in LC–MS grade acetonitrile (mobile phase B) run at a flow rate of 0.3 ml/min. Flow below 0.45 ml/min Mobile phase gradient: 0–1 min (5% B), 1–5 min (5–25% B), 5–15.5 min (25–40% B), 15.5–17.5 min (40–95% B), 17.5–19 min (95% B), 19–19.5 min (95–5% B), and 19.6–21 min (5% B). The column was maintained at 45°C and the injection volume of all samples was 5 μL. The mass spectrometer was operated in negative ion mode with a capillary voltage of 1.2kV. The source temperature is 150 °C, and the desolvation gas temperature is 550°C. The data were collected with multiple reaction monitor (MRM), and the cone and collision energy used the optimized settings from QuanOptimize application manager (Waters).

### Real-Time Quantitative PCR

Total RNA was isolated using Trizol reagent (Invitrogen, Carlsbad, CA, United States). The cDNA templates were obtained from 500 ng of the purified RNA using iScript Reverse Transcription Supermix for RT-PCR (Bio-Rad, Berkeley, CA, United States). The qPCR primers were designed and synthesized (Sangon Biotech, Shanghai, China) and the forward and reverse sequences were shown in Supplementary Table S1. Power Up SYBR Green PCR Master Mix (Applied Biosystems, ThermoFisher Scientific, Waltham, MA, United States) was used for quantitative RT-PCR, and assays were performed on an ABI Q7 PCR system (Applied Biosystems Instruments, Thermo Fisher Scientific). Targeted gene levels were normalized to housekeeping gene levels (GAPDH) and the results were analyzed using the ∆∆Ct analysis method. Data in the HFD groups were compared to the CD, HFD + TB, HFD + PCP, and HFD + TB + PCP groups (n = 8 per group).

### Hematoxylin and Eosin (H & E) Staining

The liver tissue was cut into small pieces, completely fixed with 4% paraformaldehyde solution, and then routinely dehydrated and embedded with paraffin. Sections (4 μm) of each sample were cut and histopathological analysis was performed by H&E staining. The slides were observed under light microscope. The overall magnification of the microscope is 100x (10x objective with a 10x eyepiece). There are three fields per sample. The hepatic steatosis score of H&E staining was based on the size and number of hepatic lipid droplets (Duparc et al., 2017).

### Western Blot Analysis

Liver samples were lysed on ice with RIPA buffer containing 1 mM phenylmethyl sulfonyl fluoride (PMSF) (Beyotime Technology, Shanghai, China). Total protein concentration was determined using BCA protein determination kit (Thermo, Waltham, MA, United States). Protein extract (5 μg/μL) was added to loading buffer (Beyotime Technology) and boiled at 100°C for 10 min to denature. There was 50 μg of the protein loaded per sample in western blot experiment. The equal amounts of protein were transferred to PVDF membrane by electrophoresis on 10% SDS-PAGE gel. The membrane was blocked with 5% non-fat milk and then incubated with antibodies against CYP7A1 (1:3000, ab65596, Abcam, Cambridge, MA, United States), CYP7B1 (1:3000, ab138497, Abcam, Cambridge, MA, United States), CYP8B1 (1:3000, ab191910, Abcam, Cambridge, MA, United States), CYP27A1 (1:3000, ab227248, Abcam, Cambridge, MA, United States), PPAR-α(1:3000, ab272718, Abcam, Cambridge, MA, United States), SREBP1-c (1:1500, ab28481, Abcam, Cambridge, MA, United States), CD36 (1:2000, Cat No. 18836-1-AP, Proteintech, China) and GAPDH (1:3000, Cat No. 10494-1-AP, Proteintech, China) at 4°C overnight. The membranes were washed three times by tris-buffered saline + Tween 20 (TBST) buffer and following a 1-h incubation with HRP conjugated secondary antibodies (1:3500, #7074, Cell Signalling Technology). The blots were visualized using an ECL kit (Bio-Rad, CA, United States) (n = 3 per group). The gray values of the bands were calculated using ImageJ software and were normalized to GAPDH.

### Enzyme-Linked Immunosorbent Assay (ELISA)

The levels of TNF-α, IL-6, and IL-10 in serum and liver tissue were evaluated with an ELISA kit (RK00027, RK00008 and RK00016, respectively, ABclonal, Wuhan, China) according to the manufacturer’s instructions.

### Statistical Analysis

Results were presented as mean ± SEM. All the bar plots in this study were generated by GraphPad Prism 9.0 (GraphPad Software, La Jolla, CA, United States), and differential significance analysis using the Mann–Whitney *U* test and one-way ANOVA test was performed by SPSS 26.0 (IBM SPSS, Chicago, IL, United States) followed by the post‐hoc tests (Dunnett’s test) for the normal or non‐normal distributed data, respectively with criteria as *p* value < 0.05. The sample distribution was determined using the Kolmogorov–Smirnov normality test. Metabolite classification and biomarker selection, correlation analysis, regression analysis, and pathway and enrichment analysis were performed for serum metabolism data based on the IP4M analysis previously developed by the team of the current study (Liang et al., 2020). The network map was constructed based on the data of serum metabolites analyzed and processed by the IP4M platform and directly imported into Cytoscape 3.8.2 (Cytoscape software, Santa Cruz, CA, United States).

## Results

### TB and PCP Content in Pu-erh Tea Extraction and *P. Cocos* Extraction

The average content of TB in pu-erh tea crude powder was 993 mg/g (Supplementary Table S2) and the average content of PCP in *P. Cocos* crude powder was 430 mg/g (Supplementary Table S3).

### TB and PCP Alleviated HFD-Induced Weight Gain and Hyperlipidemia

**FIGURE 1:**
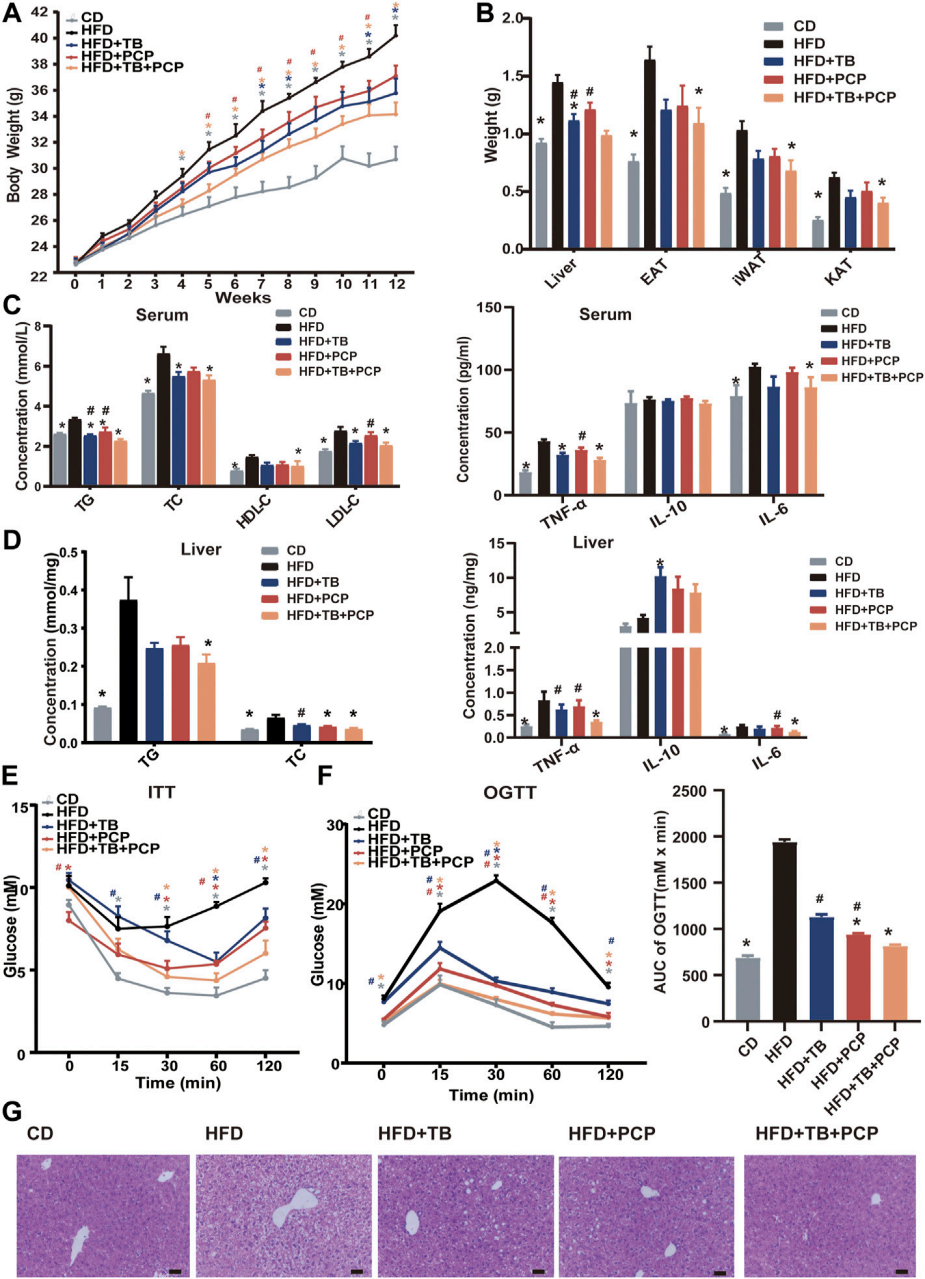
The phenotypes of the mice in CD, HFD, HFD + TB, HFD + PCP, and HFD + TB + PCP groups. **(A)** The body weights across 12-weeks treatment. **(B)** The weights of liver, epididymal adipose tissue (EAT), perirenal adipose tissue (KAT), and subcutaneous inguinal white adipose tissue (iWAT). **(C)** The levels of serum lipids (TG, TC, HDL-C, LDL-C) and inflammatory factors. **(D)** The hepatic TG and TC levels and inflammatory factors. **(E)** The blood glucose levels of ITT (baseline, and 15, 30, 60, 120 min after insulin loading). **(F)** The blood glucose levels of OGTT (baseline, and 15, 30, 60, 120 min after insulin loading) and the AUC of OGTT. **(G)** Representative images of liver HE staining. Scale bars, 50 μm. 100× magnification. Data were expressed as mean ± SEM. Differences between groups (all groups compared to the HFD group) were assessed using the one-way ANOVA test; *p < 0.05 compared with the HFD group. Differences between groups (HFD + TB and HFD + PCP compared to the HFD + TB + PCP group) were assessed using the one-way ANOVA test; # < 0.05 compared with the HFD + TB + PCP group.

To explore the therapeutic effect of TB and PCP, 8-week-old male C57BL/6J mice were randomly divided into five groups: CD group, HFD group, HFD supplemented with 0.3% TB (HFD + TB), HFD supplemented with 0.15% PCP (HFD + PCP), and HFD with 0.3% TB and 0.15% PCP (HFD + TB + PCP). After 12 weeks of treatment, the mice in the HFD group showed significantly increased body weight compared to chow diet controls. TB, PCP, and the combination of TB and PCP intervention reduced HFD-induced body weight gain (Figure 1A). Compared with the HFD + PCP group, body weights were significantly lower in HFD + TB + PCP group from the fifth to the eleventh week (Figure 1A). No difference in food intake was observed among the five groups (Supplementary Figure S1A). TB, PCP, and TB + PCP treatment lowered the weight of liver and epididymal adipose tissue (EAT), perirenal adipose tissue (KAT), and subcutaneous inguinal white adipose tissue (iWAT) (Figure 1B). Serum TG, TC, HDL-C and LDL-C were decreased after TB, PCP or TB + PCP intervention (Figure 1C). Compared with the HFD group, the level of hepatic TG was significantly lower in TB + PCP group, but not in TB group or PCP group. The hepatic TC was significantly lower in TB, PCP and TB + PCP, compared to HFD group (Figure 1D). Hepatic and the serum TNF-α and IL-6 were significantly higher in the HFD group compared with the CD group. TNF-α and IL-6 were relatively lower in the TB, PCP or TB + PCP groups. Notably, the levels of hepatic TNF-α and IL-6 were significantly lower in the TB + PCP group, which didn’t observe in the TB or PCP group (Figure 1C,D). These results showed that TB or PCP, especially the combination of TB and PCP, could alleviate the liver inflammation in the HFD mouse models. In addition, the ITT, the OGTT, and AUC of the OGTT showed that TB, PCP, and TB + PCP improved glucose tolerance and insulin resistance, among which TB + PCP was the most effective on hypoglycemia (Figure 1E,F). When comparing the phenotypes among PCP, TB and PCP + TB groups, the liver weight (Figure 1B), TNF-α in the liver (Figure 1D), TG in the serum (Figure 1C), and the AUC of OGTT (Figure 1F) were significantly lower with TB + PCP intervention compared to TB or PCP respectively. H&E staining of livers showed an obvious alleviation of hepatocyte steatosis after treatment of TB and PCP, especially TB + PCP (Figure 1G). The severity of the steatosis was scored on H&E staining liver sections based on the size and number of hepatic lipid droplets as proposed (Duparc et al., 2017). The results showed that liver lipid content was increased in HFD group and decreased in the treatment in TB, PCP, TB + PCP groups (Supplementary Figure S1C).

### TB and PCP Intervention Altered the Serum Metabolite Profiles

**FIGURE 2:**
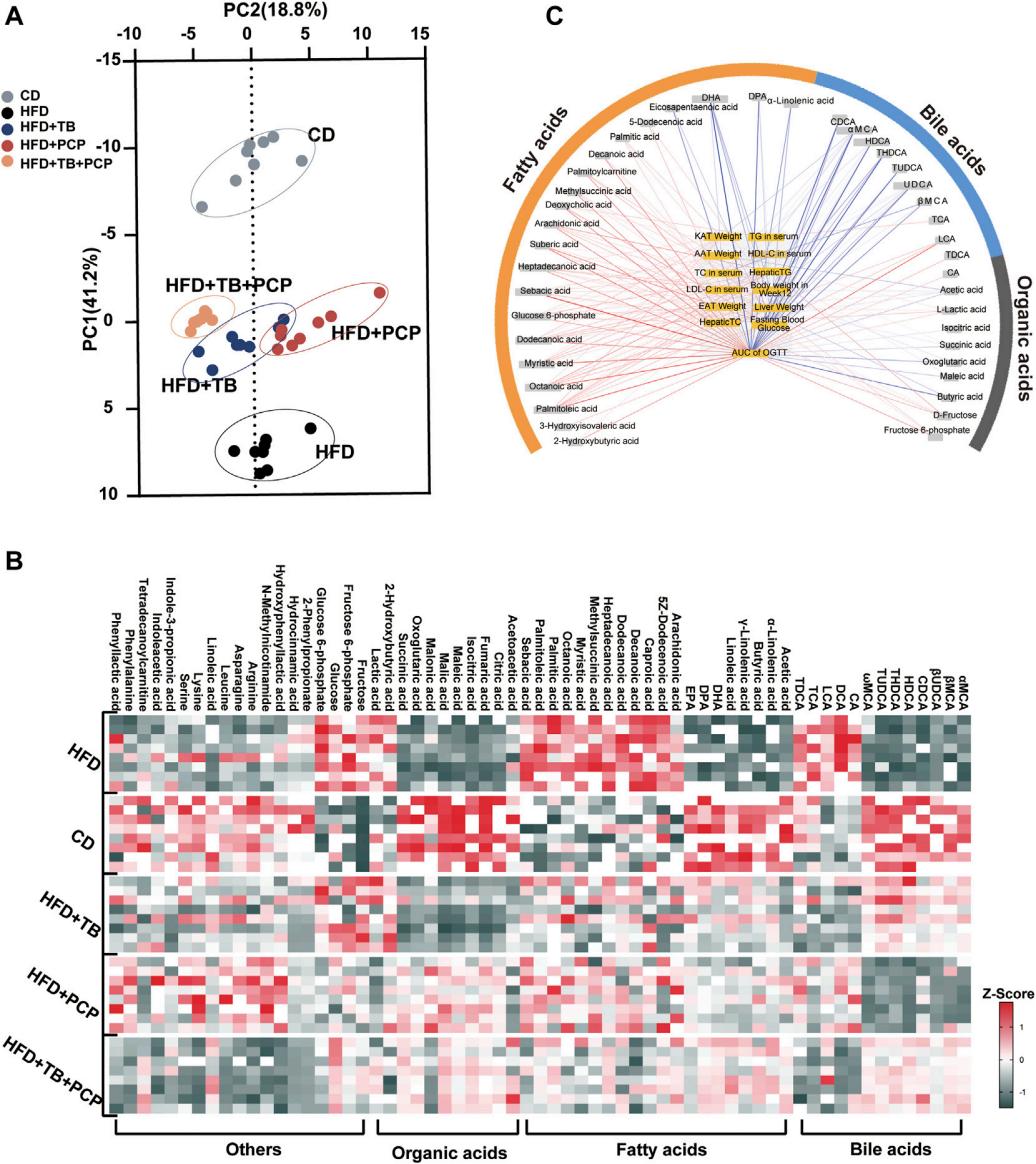
**(A)** Partial least squares discriminant analysis (PLS-DA) for CD, HFD, HFD + TB, HFD + PCP, and HFD + TB + PCP groups. **(B)** The heatmaps of serum metabolites in the five groups. The *z-*score reflects the number of standard deviations from the mean for each metabolite. **(C)** Network integrating phenotype–metabolite correlations. Phenotypes are shown in yellow and significant correlative metabolites are shown in gray. Red and blue lines represent positive and negative correlations, respectively. The thickness of the line represented the strength of the correlation.

Metabolomics analysis of the serum samples revealed significantly altered metabolites in the serum after TB, PCP, and TB + PCP interventions. The scores plot of Partial least squares discriminant analysis (PLS-DA) showed that the metabolite profiles of the HFD group were separated from the CD group. With TB, PCP, or TB + PCP interventions, the metabolite profiles were approaching the CD group, especially TB + PCP group (Figure 2A). The results revealed that TB and PCP had a recovery effect on HFD-induced metabolic changes. The heatmap of the serum metabolites showed that the most profound metabolic alterations were between the CD group and HFD groups. Compared to the CD group, the HFD group showed a higher abundance of saturated fatty acids and 12α-hydroxylated BAs. TB + PCP treatment yielded serum metabolite patterns generally closer to the CD group, and the TB or PCP treatment appeared to be intermediate (Figure 2B). The Pearson correlation analysis showed that fatty acids were positively correlated, and BAs were mainly negatively correlated to the phenotypic parameters (Figure 2C). The alteration of fatty acid and BA might be due to the lipid uptake from the intestine, *de novo* liver lipogenesis, the fluxion of excessive fatty acids into the liver from the hepatic portal vein, which can result in hepatic lipid overload and steatosis (Donnelly et al., 2005; Hodson and Gunn, 2019) (Supplementary Figure S2). Therefore, whether the observation of hyperlipidemia and hyperglycemia alleviation by TB, PCP, and TB + PCP was related to these mechanisms was further investigated.

### TB Improved Hyperlipidemia by Modulating the BA Synthesis and Fatty Acid Metabolism

**FIGURE 3:**
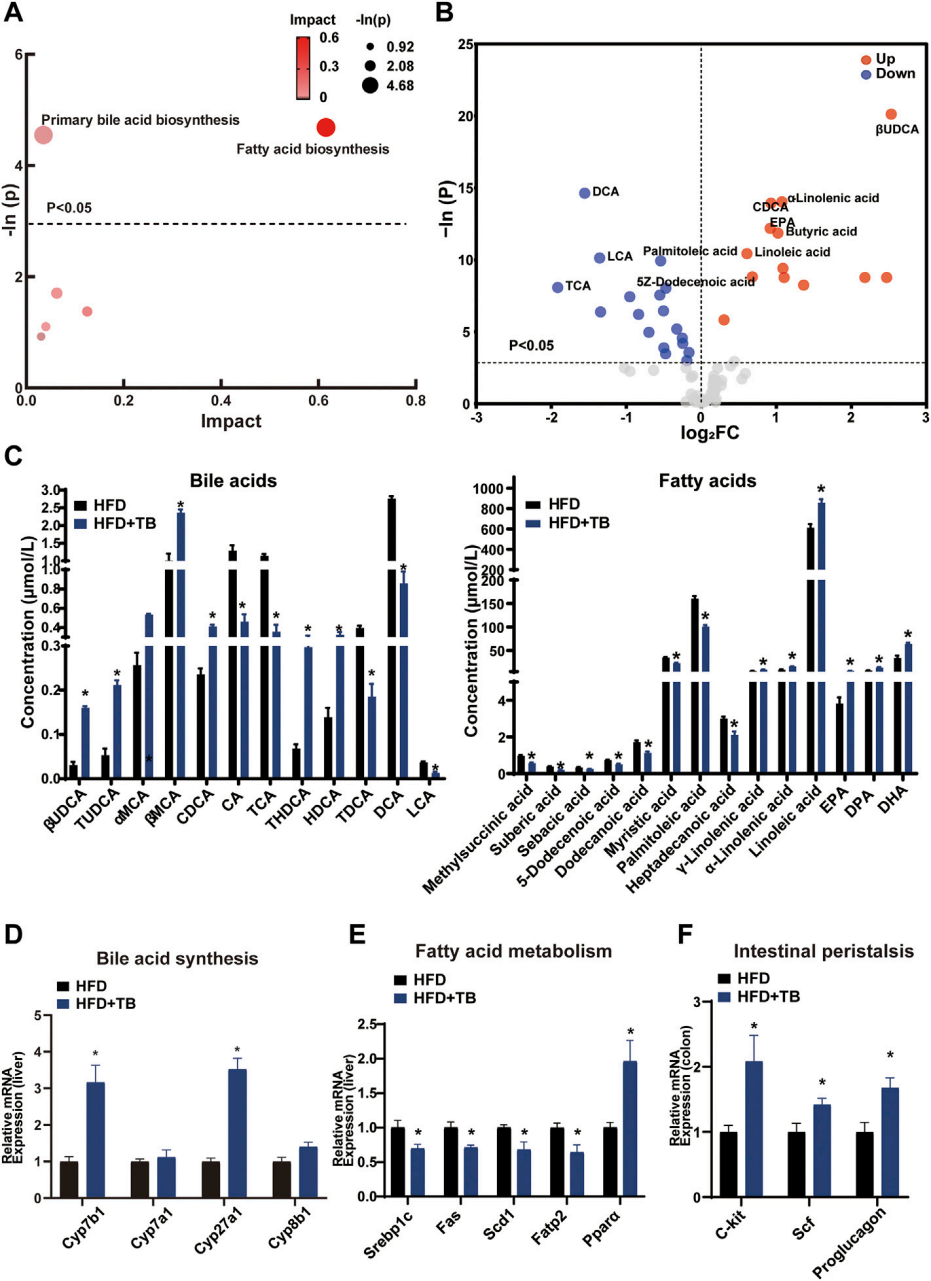
**(A)** Bubble chart of potential signaling pathways. Pathway analysis was performed in the KEGG database. The color and size of each bubble represent the impact of metabolites in each pathway and *p* value, respectively. **(B)** Volcano plot for the differential metabolites (HFD + TB group vs. HFD group). Metabolites with statistically differential levels lie above the horizontal threshold line. Metabolites with large fold change values lie far from the vertical threshold line at log2-fold change = 0, indicating whether the metabolite is up or downregulated. **(C)** The concentrations of differential serum metabolites. **(D)** The mRNA expression of hepatic BA synthetic genes. **(E)** The mRNA expression of hepatic fatty acid metabolism-related genes. **(F)** The mRNA expression of intestinal peristalsis-related genes. n = 8 individuals/group. Data were expressed as mean ± SEM. Differences between data were assessed by the Mann–Whitney *U* test; *p < 0.05 compared with the HFD group.

The KEGG pathway analysis of the differentially expressed metabolites was conducted to uncover the effect of TB on metabolic pathway alterations. After TB intervention, the metabolic pathway of the primary BA biosynthesis and fatty acid biosynthesis significantly changed in mice (Figure 3A). BAs, such as cholic acid (CA) and deoxycholic acid (DCA) with 12-hydroxy on steroid ring, decreased in the TB group, while chenodeoxycholic acid (CDCA) and β-ursodeoxycholic acid (βUDCA) as non-12-hydroxylated BAs on steroid ring increased with TB treatment. Moreover, saturated fatty acids (e.g., palmitic and 5Z-dodecenoic acids) decreased in the TB group (Figure 3B,C). The mRNA expression levels of genes involved in BA and fatty acid biosynthesis were further investigated to confirm the metabolic alterations observed in the metabolomic results. The key enzymes in the BA synthesis pathway including *Cyp7b1* and *Cyp27a1* significantly increased in the TB group, which implied that the TB intervention may promote cholesterol conversion to BA (Figure 3D). The protein expression of CYP7B1 and CYP27A1 also increased (Supplementary Figure S3A). The mRNA expression of fatty acid synthesis, desaturation, and absorption was found to be inhibited in the TB group. These enzymes included sterol regulatory element-binding proteins (*Srebp1c*) regulating the gene expression of several key enzymes in lipid synthesis, fatty acid synthase (*Fas*), stearoyl-CoA desaturase (*Scd1*) related to the desaturation of fatty acid, and fatty acid transport protein 2 (*Fatp2*) in favor of the absorption of fatty acid in the liver. The mRNA expression of fatty acid oxidation was significantly increased in the TB group such as peroxisome proliferator-activated receptor α (*Pparα*) which also increased in protein expression level (Figure 3E and Supplementary Figure S3B). In addition, several studies demonstrated that tea could promote gut motility (Sharma and Rao, 2009; Murad and Abdallah, 2014). The mRNA expression of the intestinal peristalsis-related gene including receptor tyrosine kinase (*C-kit*), stem cell factor (*Scf*), and proglucagon was found to be enhanced. The lipid transport and TCA cycle-related genes showed no significant difference in the TB group (Supplementary Figure S3C,D). Consequently, TB not only can reduce fatty acid synthesis but also promote its utilization.

### PCP Improved Lipid Metabolism by Increasing Liver Lipid Transportation to the Blood and Promoting Glucose Metabolism

**FIGURE 4:**
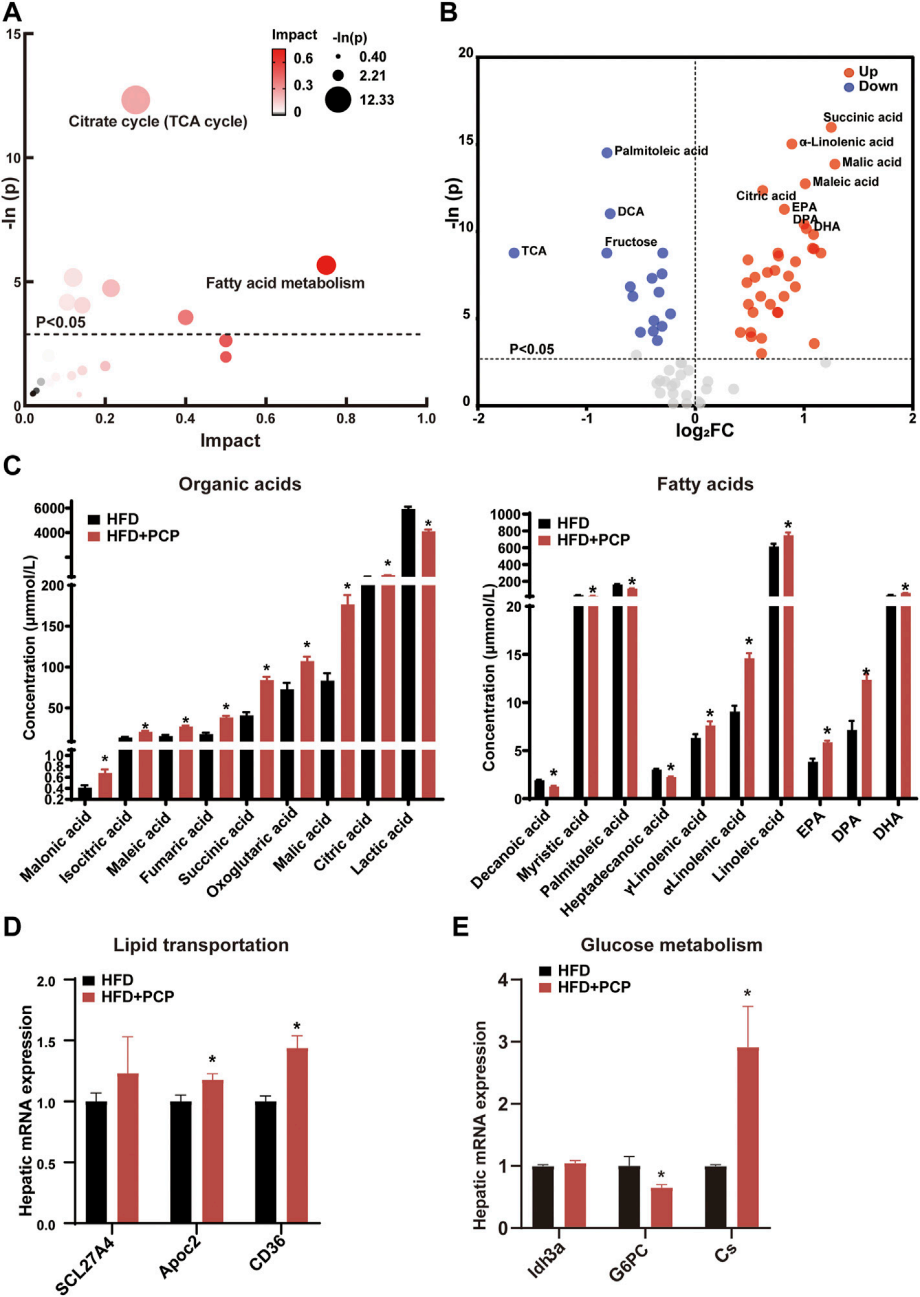
**(A)** Bubble chart of potential signaling pathways. Pathway analysis was performed in the KEGG database. The color and size of each bubble represent the impact of metabolites in each pathway and *p* value, respectively. **(B)** Volcano plot for the differential metabolites (HFD + PCP group vs. HFD group). Metabolites with statistically significant differential levels lie above a horizontal threshold line. Metabolites with large fold change values lie far from the vertical threshold line at log2-fold change = 0, indicating whether the metabolite is up or downregulated. **(C)** The concentration of differential serum metabolites. **(D)** The mRNA expression of hepatic lipid transportation-related genes. **(E)** The mRNA expression of TCA cycle-related genes. n = 8 individuals/group. Data were expressed as mean ± SEM. Differences between data were assessed by the Mann–Whitney *U* test; *p < 0.05 compared with the HFD group.

The lipid and glucose metabolism pathways (e.g., TCA cycle and fatty acid metabolism) significantly changed (Figure 4A) after PCP intervention. Following the marked reduction in the degree of liver steatosis shown by H&E staining in the PCP group, the mRNA expression of lipid transportation, such as solute carrier family 27 member 4 (*Scl27a4*), apolipoprotein C2 (*Apoc2*), and *CD36* (a fatty acid translocase), significantly increased in the PCP group (Figure 4D). The mRNA expression of lipid synthesis and desaturation (e.g., *Srebp1c* and *Scd1*) decreased compared to the HFD group (Supplementary Figure S4B). Metabolites (e.g., succinic acid and citric acid) involved in glucose metabolism increased, which were intermediate in the TCA cycle. Some lipid metabolites (e.g., palmitoleic and dodecanoic acid) decreased (Figure 4B,C). To further decipher whether PCP could influence hepatic glucose metabolism, the mRNA expression of gluconeogenesis was found to be significantly reduced, such as glucose-6-phosphatase (*G6pc*), while the mRNA expression of the TCA cycle increased including citrate synthase (*Cs*) and isocitrate dehydrogenase three catalytic subunit alpha (*Idh3α*) (Figure 4E). Different from the effect of TB, the mRNA and protein expression of BA synthesis, fatty acid metabolism, and, the mRNA expression of intestine peristalsis showed no significant difference in the PCP group (Supplementary Figure S4). These results suggested that PCP can promote lipid transportation and glucose oxidative utilization to alleviate fatty acid degeneration in the liver.

### The Improvement of Lipid Metabolism by the Combined TB and PCP Treatment

**FIGURE 5:**
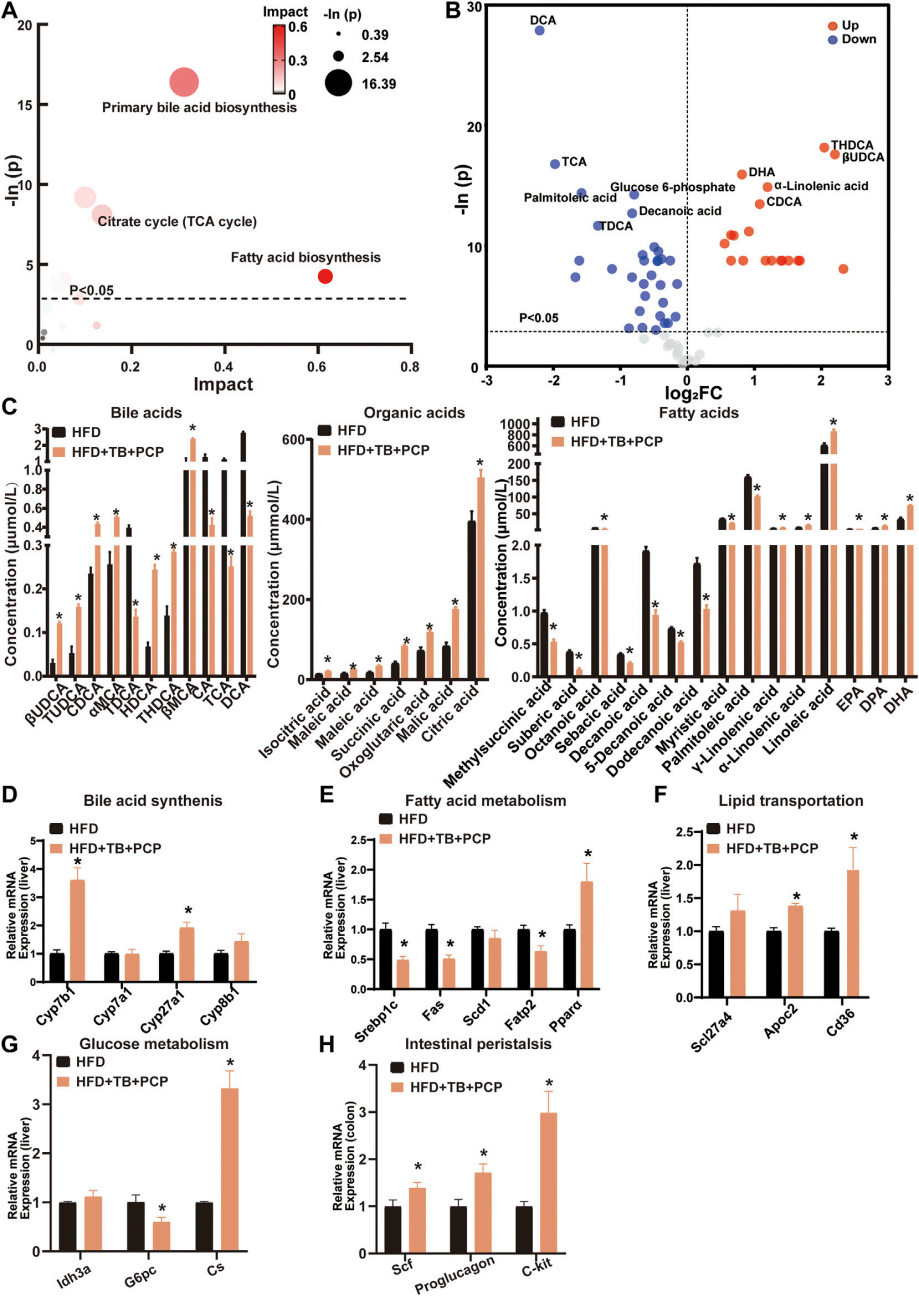
**(A)** Bubble chart of potential signaling pathways. Pathway analysis was performed in the KEGG database. The color and size of each bubble represent the impact of metabolites in each pathway and *p* value, respectively. **(B)** Volcano plot for the differential metabolites (HFD + TB + PCP group vs. HFD group). Metabolites with statistically significant differential levels lie above a horizontal threshold line. Metabolites with large fold change values lie far from the vertical threshold line at log2-fold change = 0, indicating whether the metabolite is up or downregulated. **(C)** The concentration of differential serum metabolites. **(D)** The mRNA expression of hepatic BA synthetic genes. **(E)** The mRNA expression of fatty acid metabolism-related genes. **(F)** The mRNA expression of hepatic lipid transportation genes. **(G)** mRNA expression of TCA cycle-related genes. **(H)** The mRNA expression of intestinal peristalsis-related genes. n = 8 individuals/group. Data were expressed as mean ± SEM. Differences between data were assessed by the Mann–Whitney *U* test; *p < 0.05 compared with the HFD group.

To further investigate the metabolic alterations in the HFD + TB + PCP group, the metabolites in the HFD + TB + PCP group were found to have a great impact on primary BA synthesis, fatty acid biosynthesis, and citrate cycle (Figure 5A). In addition, the HFD + TB + PCP intervention reduced the concentration of fatty acids. Non-12α-Hydroxylated BAs (e.g., CDCA and UDCA) increased while 12α-hydroxylated BAs including CA and DCA decreased (Figure 5B,C). The related genes of the BA synthesis and fatty acid metabolism were investigated, and the mRNA expression of *Cyp7b1* and *Cyp27a1* was found to be significantly elevated while the expression of fatty acid synthesis and absorption genes (e.g., *Srebp1c* and *Fatp2*) were downregulated (Figure 5D,E and Supplementary Figure S5B). Similarly, the lipid transportation genes (i.e., *Apoc2* and TCA cycle genes like *Cs*) were upregulated in the HFD + TB + PCP group (Figure 5F,G). The gene expressions related to intestinal peristalsis increased which implied that the combination of TB and PCP could accelerate gut motility (Figure 5H). Compared with PCP group, non-12α-hydroxylated BAs(non-12OH-BAs) like TUDCA significantly increased, and 12α-hydroxylated BAs(12OH-BAs) like DCA and saturated fatty acids like decanoic acid significantly decreased in the combination of TB and PCP. Moreover, the concentrations of organic acids in the tricarboxylic acid cycle such as succinic acid were higher in HFD + TB + PCP group than HFD + TB group (Supplementary Figure S6). Based on the aforementioned data, TB + PCP exhibited comprehensive lowering-lipid function, which not only exhibited the effect of inhibiting *de novo* lipogenesis, promoting cholesterol transformation into BA and fatty acid oxidation of TB but also promotes lipid transport from the liver and TCA cycle of PCP.

## Discussion

The effect of TB + PCP on reducing lipid has not been explained although previous studies revealed that TB and PCP could alleviate hyperlipidemia through several mechanisms (Huang et al., 2019a; Huang et al., 2019b; Kim et al., 2019; Kuang et al., 2020). In the current study, the combination of TB and PCP was found to significantly reduce body weight, adipose tissue weight, and lipid levels (e.g., TG, TC, and LDL in the liver). The TB and PCP combination changed serum metabolites, which improved lipid and glucose metabolism.

Lipid overload in the liver causes great injury to the liver including inflammatory and liver cell injury which can ultimately lead to liver cirrhosis (Byrne and Targher, 2015; Friedman et al., 2018; Li et al., 2022). The hepatoprotective function of natural herbs by ameliorating hyperlipidemia is verified in clinical trials (Yan et al., 2020) which exhibit therapeutic effects on fatty liver. Therefore, the exploration of natural products to alleviate lipid disorder is promising and necessary. A previous study has shown that pu-erh tea can change the composition of gut bacteria that produces a difference in BA metabolism, in which gut microbiota facilitated the metabolism of primary to secondary BAs in the gastrointestinal tract after TB intervention. In a gut microbiota-dependent manner, TB promoted energy expenditure in adipose tissue and improved lipid metabolism. The mRNA expression of *Cyp7b1* and *Cyp27a1* was enhanced which promoted the alternative synthesis pathway of BA (Huang et al., 2019b). The gut microbiota plays an important effect in bile acid metabolism that some of the bacteria in the gut express the key enzymes of bile acid metabolism such as bile-salt hydrolase (BSH) and 7-α dehydroxylase, facilitating the deconjugation and dehydroxylation in the process of bile acid metabolism. Our previous study has proved that TB can change the composition of gut bacteria involved in BA metabolism (Huang et al., 2019b). TB suppressed BSH enriched bacteria including *Lactobacillus*, *Bacillus*, *Enterococcus*, Lactococcus, *Streptococcus*, and Leuconostoc genera in mice, which increased the conjugated bile acids like TUDCA (Huang et al., 2019b). We also found that TB could elevate the abundance of 7α-dihydroxylation-enriched microbes like *Clostridium* scindens and Parabacteroides distasonis, and increase non-12OH BAs in feces (Kuang et al., 2020). In this study, the composition of bile acids was greatly changed after TB and TB + PCP intervention with increased levels of the non-12OH BAs such as βUDCA, TUDCA and THDCA, which implied that TB or TB + PCP could regulate the composition of gut microbiota. TB was reported to expedite energy metabolism in adipose tissue and promote the transformation of white adipose to brown adipose (Kuang et al., 2020). Hence, TB is beneficial to reduce hepatic steatosis. In this study, BA metabolites (e.g., βUDCA and CDCA) were found to be elevated in the serum, and the mRNA expression of BA synthesis-related gene was increased, which promoted cholesterol transformation into BAs in the liver with TB intervention. Several pathways contribute to the fatty liver including the absorption of fatty acid from the diet and the *de novo* lipogenesis in the liver (Donnelly et al., 2005). Therefore, preventing the absorption of fatty acid from the serum and the synthesis of fatty acid can effectively lessen steatohepatitis. In this study, TB constrained the mRNA expression of fatty acid absorption and synthesis-related genes and promoted fatty acid oxidation. These results suggested that TB may have a therapeutic effect on the fatty liver by increasing BA biosynthesis and fatty acid depletion while, in turn, inhibiting fatty acid synthesis. *Supplement to compendium of materia medica* written by famous Chinese medical scientist Zhao Xuemin in the Eastern Qing Dynasty explained that pu-erh could promote digestion and intestinal peristalsis. Consistently, the mRNA expression of related genes (e.g., *C-kit* and *Scf*) was found to be enhanced.

PCP is one of the most effective ingredients of *P. cocos* and has been applied in clinical treatment to improve hyperlipidemia (Brunt et al., 2015; Miao et al., 2016) and hyperglycemia (Xie and Du, 2011). Most plant polysaccharides are difficult to be absorbed in the intestinal tract (Xia et al., 2021). PCP alleviates lipid metabolism disorder mainly via modulating the gut microbiota composition and the microbial metabolites (Sun et al., 2019a; Wang et al., 2019; Sun et al., 2020). Previous study has shown that PCP changes the feces microbial structure and composition, which enhance the microbial richness and diversity (Wang et al., 2019). Our data showed that PCP intervention significantly changed BA pool and composition. BAs, produced in the liver, are metabolized by enzymes derived from intestinal bacteria, so that glyco-conjugated and tauro-conjugated BAs are deconjugated via BSH and 7α-dehydroxylated to form secondary BAs (Jia et al., 2018; Xie et al., 2021b). The gut microbiota and BA interaction are critically important for maintaining the function of intestinal motility and permeability (Jia et al., 2018), balanced lipid and carbohydrate metabolism (Jia et al., 2021), insulin sensitivity (Zhang et al., 2019) and innate immunity (Jia et al., 2018). Meanwhile, with the action of bacteria, PCP transforms into short-chain fatty acids such as butyrate found in our study. These metabolites are beneficial to intestinal integrity and promotes β-oxidation by activating the peroxisome proliferator-activated receptor-gamma signaling (Sun et al., 2019a; Sun et al., 2020). Some reports also showed that PCP could prevent the overgrowth of gut fungi which induces proinflammatory factors in liver and results in hepatic fat accumulation (Henkel et al., 2018; Sun et al., 2020). Glucose is one of the main precursors of lipid synthesis, and superfluous glucose can lead to hyperlipidemia and hyperglycemia (Wang et al., 2020). In this study, the mRNA expression of glucose expedition-related genes (e.g., *Cs* and *Idh3a*) was upregulated, and the glycogenesis-related gene was downregulated, suggesting that PCP may be conducive to reducing blood glucose. Moreover, PCP may improve steatohepatitis through accelerating lipid transportation from the liver to the serum. Thus, PCP intervention upregulated the expression of transportation proteins that promote lipid transport to circulation and adipose tissue.

**FIGURE 6:**
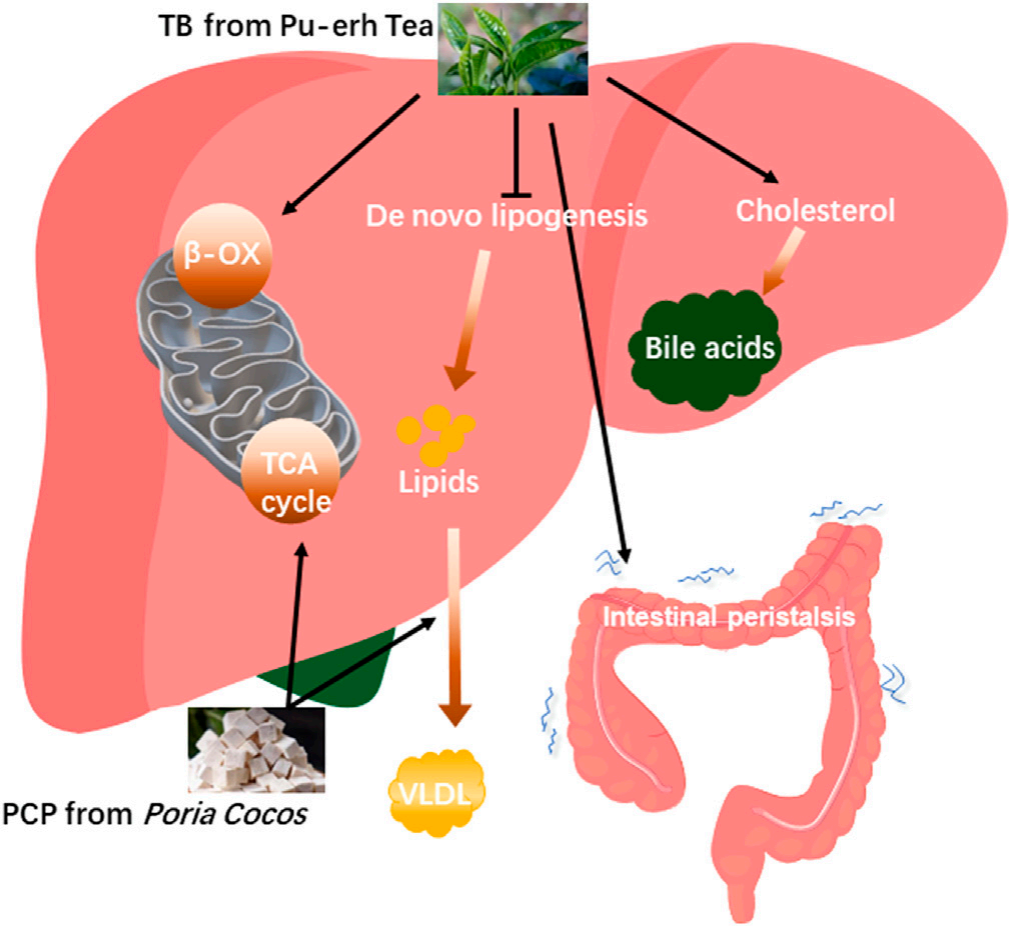
The proposed mechanisms of theabrownin and *P. cocos* polysaccharide on the NAFLD alleviation.

In TCM, the formula for treatment consists of multiple herbs which will make the treatment effect more remarkable (Wang et al., 2021). The TCM compatibility rationale considers that different parts of the disease can be simultaneously intervened to achieve the best therapeutic effect with the combination of different herbs (Zhou et al., 2017). One study demonstrated that natural products including white peony root and licorice as well as grape seeds and broccoli extracts ameliorate hepatic steatosis and glucose tolerance (Chen et al., 2021b) which represented that the combination of natural products improved NAFLD. In this study, TB combined with PCP to alleviate fatty liver has been explored based on a previous study about TB and PCP in NAFLD treatment. TB + PCP was found to promote cholesterol BA formation while reducing fatty acid synthesis, reuptake, and oxidative utilization and promoting the oxidation and utilization of glucose and the hepatic lipid transport, which efficiently ameliorate hepatic excessive lipid accumulation. Bile acid pool diversity is attributed to a collaborative metabolism of the host and gut microbiota. NAFLD is characterized by an altered composition of bile acids. Some bile acids are accumulated in the liver and have hepatotoxic potential, while some are beneficial to the liver and even have therapeutic effect on hepatic lipid accumulation and inflammation (González et al., 2011; Sun et al., 2019b). In this study, we found non-12OH-BAs such as CDCA and TUDCA were significantly increased in TB and TB + PCP groups, and CDCA was negatively correlated with TC and TG levels. UDCA was reported to improve hepatic steatosis by reducing circulating fibroblast growth factor 19 and blunting farnesoid X receptor activation, which effectively alleviated hepatic steatosis (Mueller et al., 2015). In the meanwhile, 12OH-BAs such as CA and DCA were decreased in TB and TB + PCP groups, which were reported to promote lipogenic gene expression stearoyl-CoA desaturase 1 (SCD1), including, fatty acid synthase (FAS) and sterol regulatory element-binding protein 1 (SREBP-1) (Jia et al., 2021). Bodyweight and the weight of adipose tissue and liver in the HFD + TB + PCP group were observed to be the lowest in the last week and the body weight of HFD + TB + PCP was significantly decreased in the earliest compared to TB, PCP respectively. Compared with the HFD group, the weight of EAT, KAT and iWAT were significantly lower in TB + PCP group, but not in TB group or PCP group which implied the combination of TB and PCP might be more effective in improving lipid metabolism than TB or PCP respectively. The reduction of TC and TG in the liver and serum also had a significant effect compared to the respective TB or PCP intervention. In summary, TB was found to promote cholesterol to transform to BA and oxidize fatty acid and inhibit *de novo* lipogenesis. PCP facilitated the lipid transportation from the liver to the peripheral blood circulation and may accelerate the TCA circle in the mitochondria. In addition, TB + PCP can promote cholesterol synthesis of fatty acid, promote lipid transport and oxidative utilization, and reduce lipid synthesis (Figure 6). Thus, incorporating TB and PCP has the potential to alleviate NAFLD.

This study has some limitations that need further exploration (e.g., the effect of lipid metabolism in other tissues and organs other than the liver). In this study, a concentrated combination of TB and PCP was designed and the positive effect of alleviating fatty liver was demonstrated. However, whether different concentrate combinations will have different therapeutic effects needs further study. Besides, in this study we used male mice models only to study the therapeutic effect of TB and PCP, which was a commonly used model for NAFLD study in most publications. Nevertheless, estrogen also plays an important role in regulating energy balance and insulin sensitivity which is closely related to lipid metabolism (Mauvais-Jarvis et al., 2013) and it is necessary to apply both male and female models in the further study.

## Data Availability

The original contributions presented in the study are included in the article/Supplementary Material, further inquiries can be directed to the corresponding authors.
