# Supplementary material for: Theabrownin and Poria cocos Polysaccharide Improve Lipid Metabolism via Modulation of Bile Acid and Fatty Acid Metabolism
Source: Front Pharmacol. 2022 Jun 27;13:875549. doi: 10.3389/fphar.2022.875549 (PMC9271858; doi:10.3389/fphar.2022.875549)
Supplement: Supplementary file 1 [file DataSheet1.pdf]

Table S1. Primers for qRT-PCR.

Gapdh	Forward: AGGTCGGTGTGAACGGATTTG<br>Reverse: GGGGTCGTTGATGGCAACA
Pparα	Forward: CCCAAGGGAGGAATAGCTTCT<br>Reverse: CTCTGCGATGCGGTTCCAA
Srebp1C	Forward: GGAGCCATGGATTGCACATT<br>Reverse: GCTTCCAGAGAGGAGGCCAG
Fas	Forward: AAGTTGCCCGAGTCAGAGAACC<br>Reverse: ATCCATAGAGCCCAGCCTTCCATC
Scd1	Forward: TTCTTGCGATACACTCTGGTGC<br>Reverse: CGGGATTGAATGTTCTTGTCGT
Fatp2	Forward: CGAGACGAGACGCTCACCTA<br>Reverse: ACGAATGTTGTAGTTGAGGCAC
Slc27a5	Forward: CTACGCTGGCTGCATATAGATG<br>Reverse: CCACAAAGGTCTCTGGAGGAT
Apoc2	Forward: GATGTTGGGAAATGAGGTCCAG<br>Reverse: CCTTGGCAGAGGTCCAGTAAC
Cd36	Forward: GATTAATGGCACAGACGCAGC<br>Reverse: CAGATCCGAACACAGCGTAGA
Cs	Forward: GGACAATTTTCCAACCAATCTGC<br>Reverse: TCGGTTCATTCCCTCTGCATA
Idh3a	Forward: TGGGTGTCCAAGGTCTCTC<br>Reverse: CTCCCACTGAATAGGTGCTTTG
G6pc	Forward: CGACTCGCTATCTCCAAGTGA<br>Reverse: GTTGAACCAGTCTCCGACCA
Scf	Forward: TGACCTCGTGGCATGTATGG<br>Reverse: GGACTTTGCGGCTTTCCTATTAC
Proglucagon	Forward: TGAGATGAGCACCATTCTGGA<br>Reverse: TCCGCAGAGATGTTGTGAAGA
Cyp7a1	Forward: GCTGTGGTAGTGAGCTGTTG<br>Reverse: GTTGTCCAAAGGAGGTTCACC
Cyp7b1	Forward: GGAGCCACGACCCTAGATG<br>Reverse: GCCATGCCAAGATAAGGAAGC
Cyp8b1	Forward: CACGGGGATGTCTTCACGG<br>Reverse: TGAGCACCAGTTCTTTTGCATAG
Cyp27a1	Forward: GCACAGGAGAGTACGGAGG<br>Reverse: CGGGCAAGTGCAGCACATA

C-kit	Forward: ATCAGGGCGACTTCAATTACGA<br>Reverse: TGCTGGTGTTCAGGTTTAGGGT

Table S2. The TB content in pu-erh tea extraction.

Compound(mg/g)	Pu-erh tea extraction-1	Pu-erh tea extraction-2	Pu-erh tea extraction-3
TB	991	995	994

Table S3. The PCP content in P. cocos extraction.

Compound(mg/g)	P. Cocos extraction-1	P. Cocos extraction-2	P. Cocos extraction-3
PCP	433	430	428

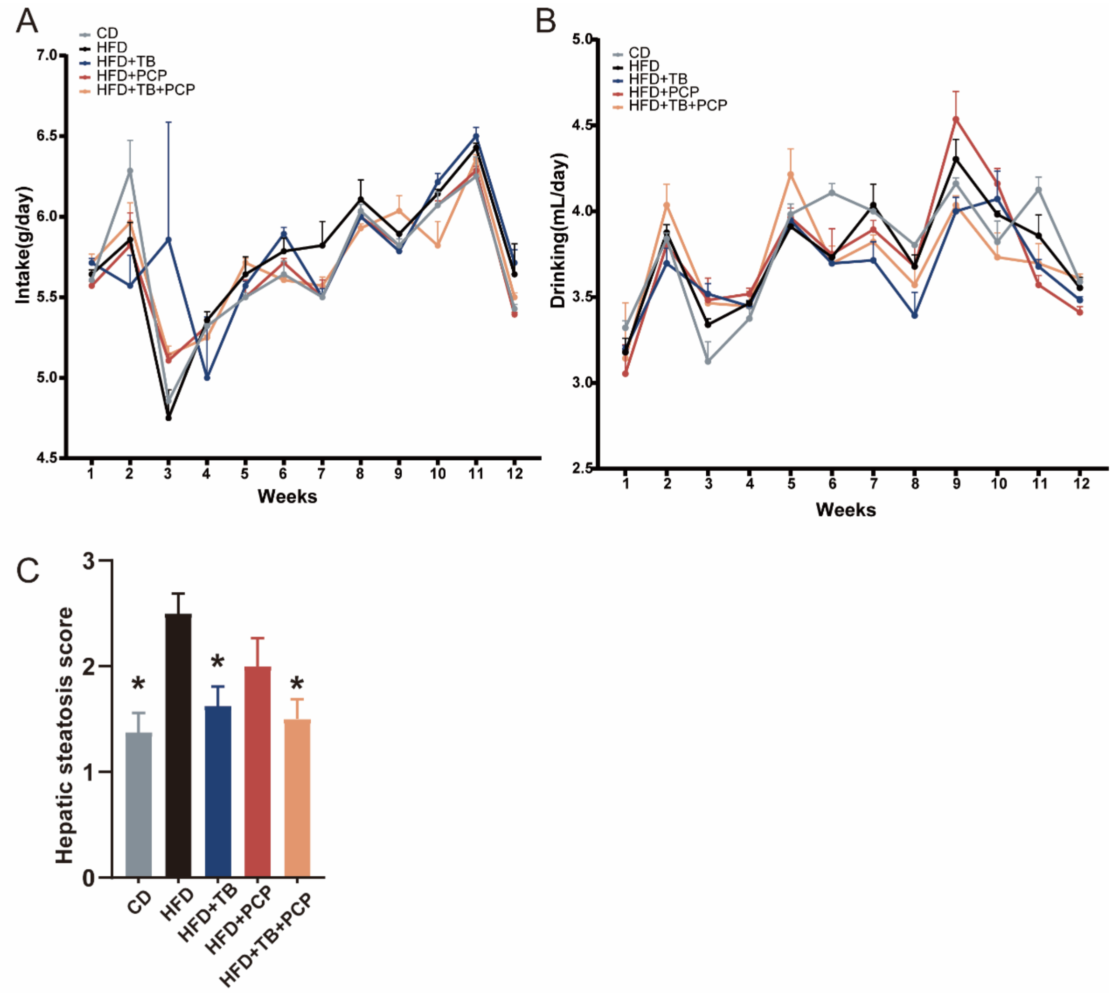

Figure S1. (A) The daily food intake of mice for 12 weeks in all groups. n= 8 per group. (B) The daily drinking of mice for 12 weeks in all groups. n= 8 per group. (C) Hepatic steatosis score in all groups. n= 3 per group. Data in the HFD groups were compared to CD, HFD + TB, HFD + PCP, and HFD + TB + PCP groups. Data were expressed as mean ± SEM. Differences between groups (all groups compared to the HFD group) were assessed using the one-way ANOVA test.

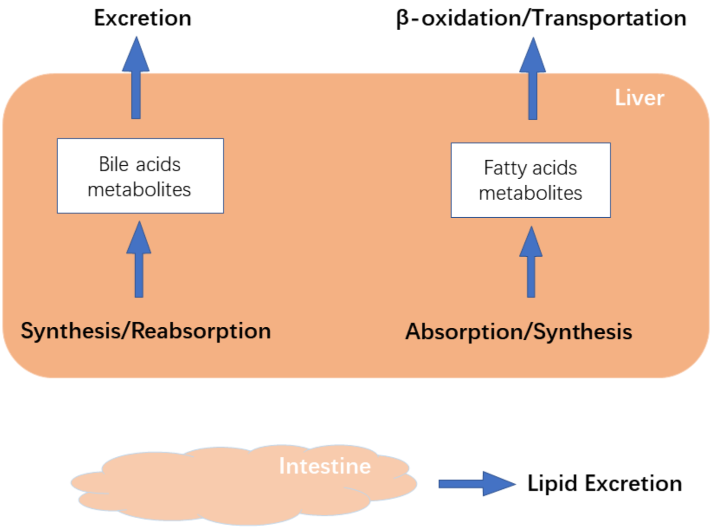

Figure S2. Proposed mechanism of lipid metabolism in mice.

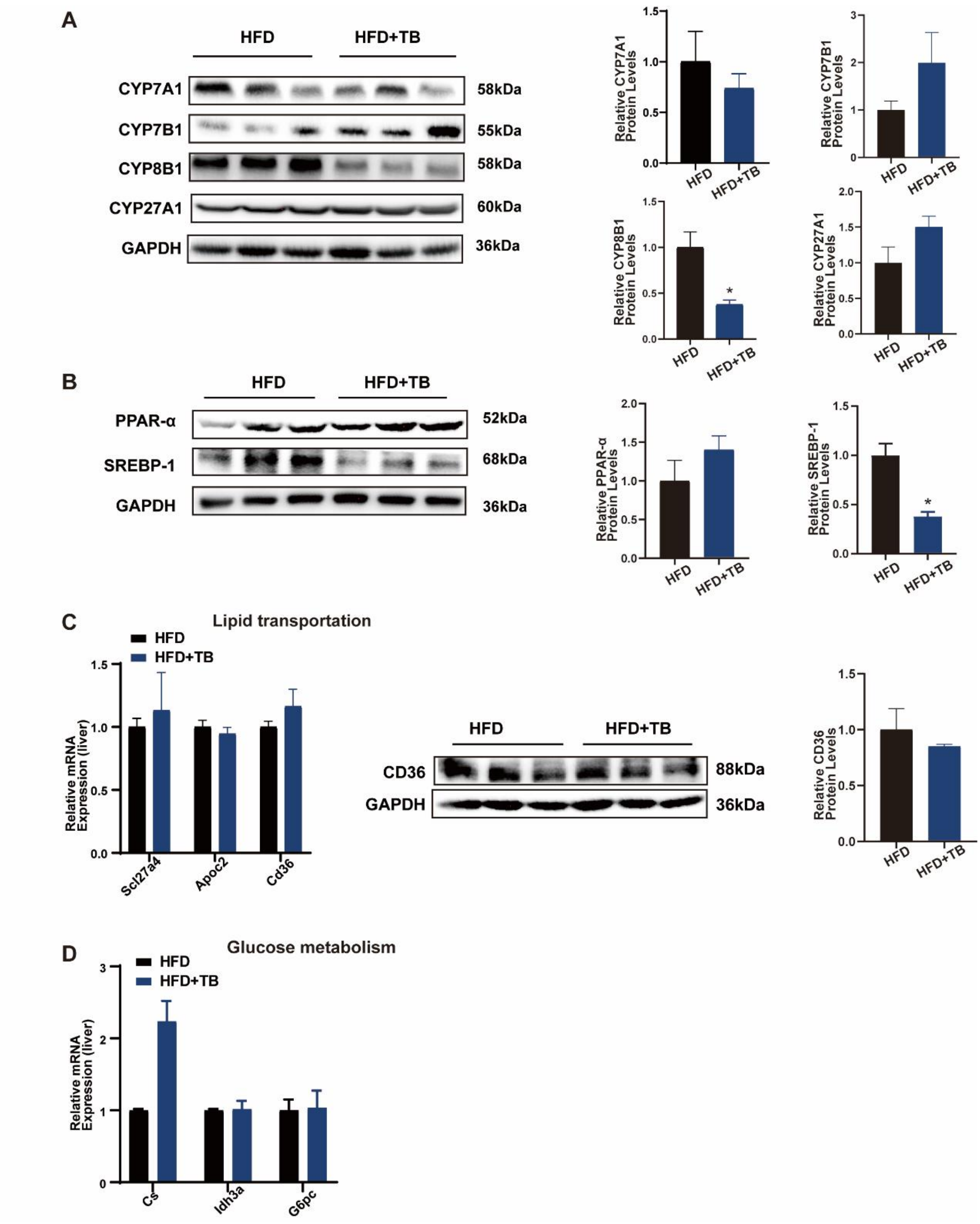

Figure S3. (A) The hepatic protein expression of bile acid synthesis related gene in the mice from HFD and HFD+TB groups. (B) The hepatic protein expression of PPAR-α and SREBP-1 in the mice from HFD and HFD+TB groups. (C) The hepatic mRNA and protein expression of CD36 in the mice from HFD and HFD+TB groups. (D) The hepatic mRNA expression of glucose metabolism in the mice from HFD and HFD+TB groups. Data were expressed as

mean ± SEM. Differences between data were assessed by the Mann–Whitney U test; $*p < 0.05$ compared with the HFD group.

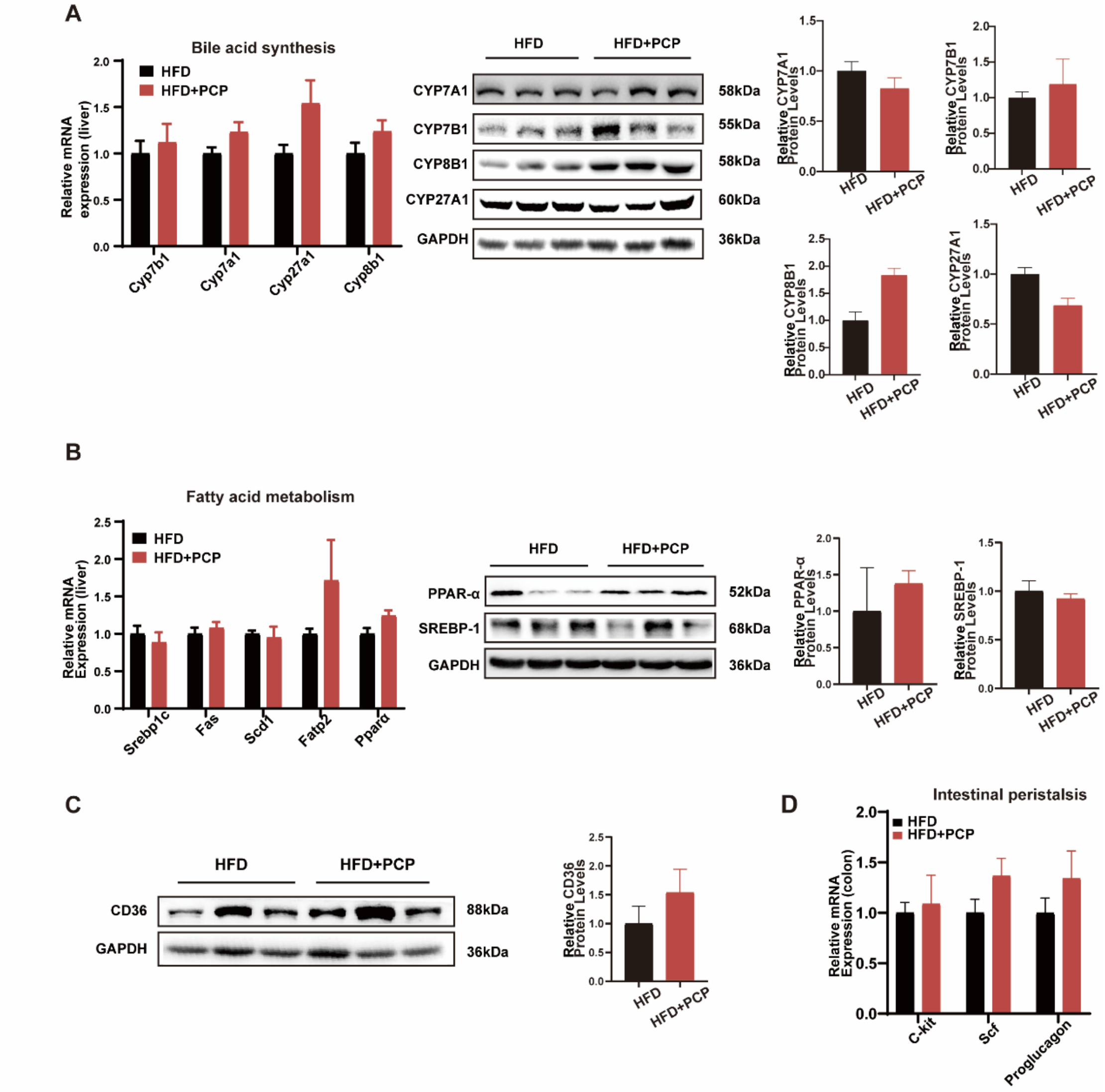

Figure S4. (**A**) The hepatic mRNA and protein expression of bile acid synthesis related genes in the mice from HFD and HFD+PCP groups. (**B**) The mRNA and protein expression of hepatic fatty acid metabolism related genes in the mice from HFD and HFD+ PCP groups. (**C**) The hepatic protein expression of CD36 in the mice from HFD and HFD+ PCP groups. (**D**) The mRNA expression of intestinal peristalsis related genes in the mice from HFD and HFD+ PCP groups. Data were expressed as mean ± SEM. Differences between data were assessed by the Mann–Whitney U test; *$p < 0.05$ compared with the HFD group.

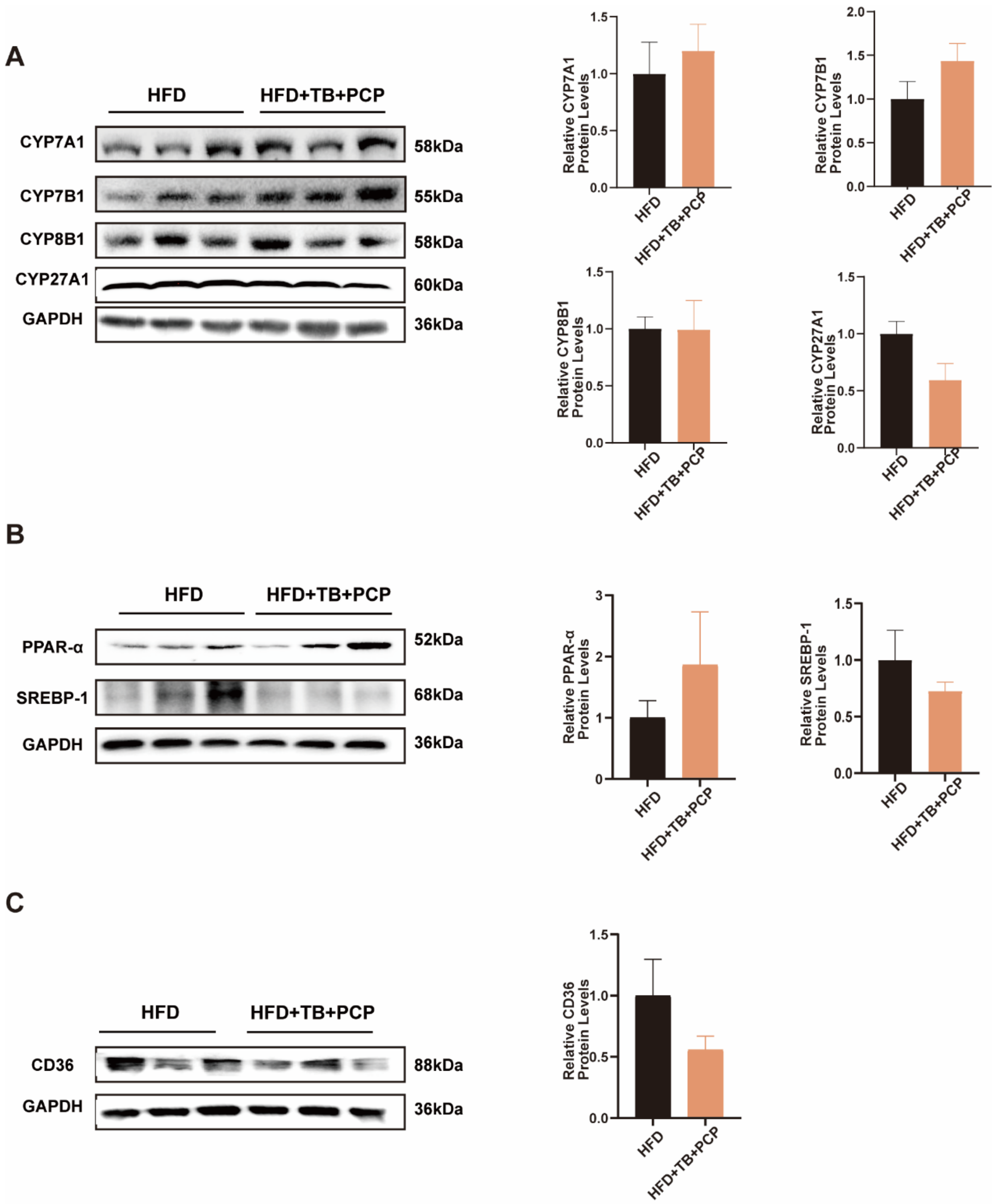

Figure S5. (A) The protein expression of bile acid synthesis-related genes in the HFD and HFD+TB+PCP groups. (B) The protein expression of fatty acid metabolism-related genes in the HFD and HFD+TB+PCP groups. (C) The protein expression of CD36 in the HFD and HFD+TB+PCP groups. Data were expressed as mean ± SEM. Differences between data were assessed using the Mann–Whitney U test; *p < 0.05 compared with the HFD group.

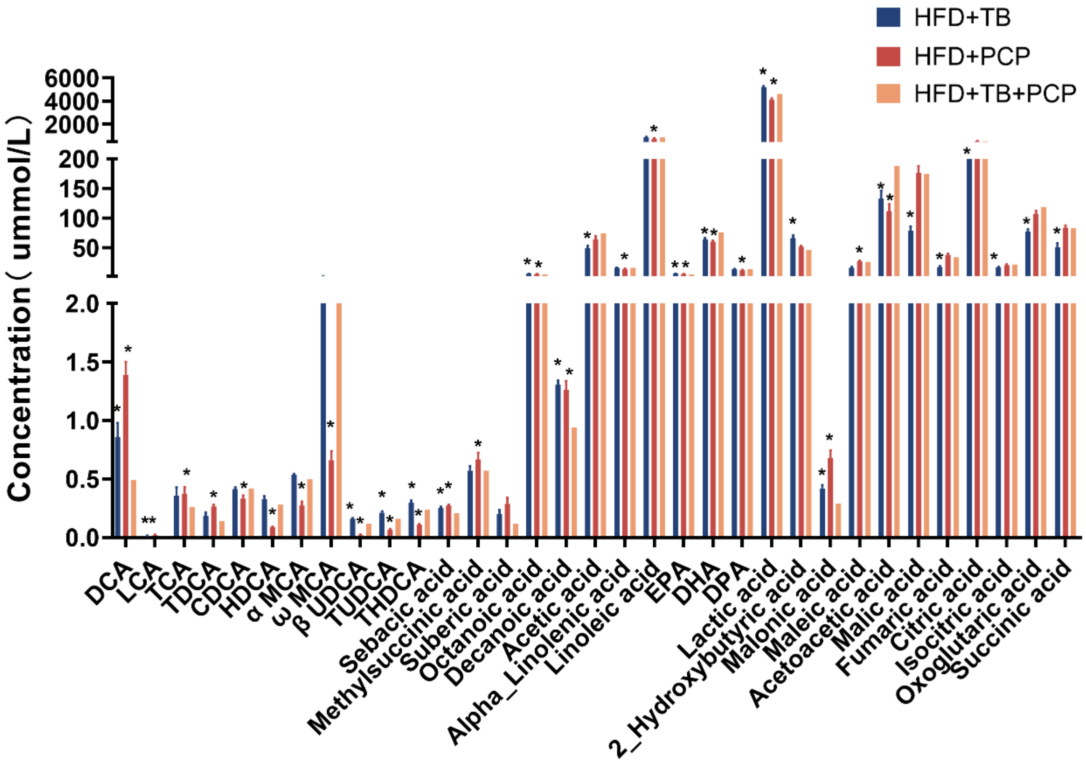

Figure S6. The concentration of serum metabolites. n = 8 individuals/group. Data were expressed as mean ± SEM. Differences between groups (HFD+TB and HFD+PCP compared to the HFD+TB+PCP group) were assessed using the one-way ANOVA test; *p < 0.05 compared with the HFD group.
